# Nitrogen use efficiency and yield gains from *Stylosanthes guianensis* integration in upland rice: insights from a ^15^N-labelling study under conservation agriculture

**DOI:** 10.1007/s10705-025-10458-w

**Published:** 2025-12-08

**Authors:** Oliver Zemek, Astrid Oberson, Julie Dusserre, Eric Scopel, Emmanuel Frossard

**Affiliations:** 1 Institute of Agricultural Sciences, ETH Zurich, Eschikon 33, 8315 Lindau, Switzerland; 2 CIRAD, UPR AIDA, TA B-115/02, Avenue Agropolis, 34398Cedex 5 Montpellier, France

**Keywords:** Madagascar, Relay cropping, Nitrogen recovery, ^15^N isotope labelling, Ferralsol

## Abstract

Efficient nitrogen (N) use remains a key challenge for upland rice production on weathered tropical soils, particularly in low-input smallholder systems where external inputs are limited and nutrient cycling is constrained. This study assessed the effects of integrating the legume *Stylosanthes guianensis* (stylo) into a conservation agriculture (CA) rotation on rice N use efficiency (NUE), and biomass productivity in Madagascar’s Midwest Highlands. A factorial field experiment tested soil tillage, stylo biomass management (retention, removal, incorporation), and N fertilizer types (none, farmyard manure, mineral fertilizer), using ^15^N isotope labelling techniques, which allowed us to quantify N sources and recovery pathways with high resolution. Mineral fertilizer resulted in the highest grain yield (429 g DM m^−2^), N uptake (6.1 g N m^−2^), and ^15^N recovery (52%), surpassing global NUE benchmarks. In the absence of fertilizer, rotation with stylo increased grain yield by 53% and N uptake by 43% compared to continuous rice (189 g DM m^−2^; 2.3 g N m^−2^), reflecting strong biological and structural legacy effects. Although stylo mulch had a high C:N ratio and contributed minimally to direct N uptake, it improved soil moisture and likely promoted conditions favourable to N mineralization. Tillage effects on N uptake and yield were negligible. These findings demonstrate that legume integration under CA can improve N cycling, enhance fertilizer N recovery, and significantly increase NUE and yield resilience in smallholder systems. Future strategies should explore how partitioning legume biomass between soil cover and fodder affects nutrient flows, and whether livestock-mediated recycling can help close N loops in mixed farming systems.

**Supplementary Information:**

The online version contains supplementary material available at 10.1007/s10705-025-10458-w.

## Introduction

Efficient nitrogen (N) use is central to achieving sustainable agricultural intensification (Govindasamy et al. 2023). While industrialized systems focus on reducing N-related environmental impacts, smallholder farming in sub-Saharan Africa (SSA) is constrained by limited access to mineral N fertilizers due to low purchasing power, poor infrastructure, and high prices (Chianu et al. 2012; Hebebrand and Laborde 2023). Combined with declining soil fertility, this makes N supply largely dependent on organic inputs and their mineralization (Dawson et al. 2008).

Conservation agriculture (CA), defined by minimal soil disturbance, permanent soil cover, and crop diversification, has gained traction as a strategy to sustain yields under resource-limited conditions (Hobbs et al. 2008; Scopel et al. 2013). Most studies in low-input systems have focused on maize-based CA systems combined with grain legumes (Corbeels et al. 2020; Pittelkow et al. 2015; Thierfelder and Wall 2011). These studies highlight that the three core CA principles can each influence N dynamics in distinct but complementary ways. Permanent soil cover with organic residues enhances microbial activity and moisture retention, promoting N mineralization; however, high C:N ratio mulches may also cause temporary N immobilization (Corbeels et al. 2020; Giller et al. 2015). Reduced tillage alters soil structure and N turnover. While it can initially slow N mineralization due to cooler and denser upper soil layers, it improves N cycling over time by conserving soil moisture and fostering microbial activity—particularly when combined with mulching (Ranaivoson et al. 2019). Tillage effects are highly context-dependent and can vary by soil type and climate (Giller et al. 2015). Crop diversification with legumes enhances soil N supply through biological N₂ fixation (BNF) and supports soil health via improved soil structure, microbial activity, and pest suppression, while reducing the need for mineral N fertilizer inputs (Franke et al. 2018; Vanlauwe et al. 2019).

Despite progress in understanding how CA principles affect N dynamics under low-input conditions, several key knowledge gaps remain. First, while the principles of CA are well established, the mechanistic understanding of how its components—particularly legume integration, residue retention, and reduced tillage—interact to influence N dynamics and crop N use efficiency (NUE) remains limited (Franke et al. 2018; Giller et al. 2015). Second, research has disproportionately focused on maize-based systems, with upland rice receiving far less attention, despite its importance in many low-input tropical environments. Specifically, the effects of legume rotation on rice NUE beyond simple yield responses are poorly documented (Asai et al. 2021; Yao et al. 2025). Third, most field studies are short-term, lack systematic N tracing, and fail to capture the context-specific interactions between CA practices, environmental variables, and fertilizer inputs that shape system performance over time (Brouder and Gomez-Macpherson 2014; Delgado et al. 2021). A comprehensive review by Grahmann et al. (2014) emphasized the importance of optimizing N inputs and enhancing NUE under CA through integrated nutrient strategies, yet highlighted the need for crop- and context-specific field validation—particularly in non-maize systems.

Nitrogen use efficiency is a key performance indicator in crop production, reflecting the plant's ability to acquire and utilize N for biomass and yield formation. It comprises two components: N uptake and recovery, *i.e.*, the amount of N absorbed from soil and fertilizers, and the physiological N utilization efficiency, *i.e.*, the conversion of absorbed N into biomass or grain (Lee 2021; Moll et al. 1982). In upland cereal systems, average fertilizer N recovery rates range between 40–50% (Ladha et al. 2005; Liu et al. 2023), while the physiological utilization efficiency optimum for modern rice varieties is about 68 g grain per g N taken up (Dobermann and Fairhurst 2000). Improving N uptake and recovery is desirable, but in low-input systems with minimal N replenishment, it can deplete soil reserves over time (Ortiz-Monasterio et al. 2001). Enhancing physiological N utilization efficiency, *e.g.*, via cultivar traits, may stabilize yields under limited inputs, although this can reduce grain N concentrations—potentially affecting nutritional quality (Vinod and Heuer 2012).

Previous work by Zemek et al. (2018) showed that *Stylosanthes guianensis* (stylo) can fix atmospheric N and improve soil N availability in a low-input CA-based legume–rice rotation in Madagascar. Unlike most CA studies, which focus on maize–grain legume systems, this research examined the less explored but highly relevant rice–legume context, where livestock in many smallholder systems creates competition for biomass use as mulch or fodder. In our study, the rice cultivar was NERICA 4, widely grown by local farmers and chosen for its suitability to marginal upland soils, relatively low P requirements, and high N responsiveness under limited P supply (Mghase et al. 2011; Oikeh et al. 2008; Sahrawat 2000). Stylo was selected for its anthracnose resistance, drought tolerance (Amezquita et al. 1991; Fisher and Ludlow 1984), capacity to provide soil cover and prevent erosion, value as livestock fodder (Chakraborty 2004), weed suppression (Michellon et al. 2011), ability to fix N**₂** under adverse conditions (Edye et al. 1984), deep rooting and nutrient recycling (Gathumbi et al. 2003), and efficient P acquisition (Li et al. 1997). Although Zemek et al. (2018) quantified stylo’s BNF contributions (96–122 kg N ha⁻^1^ over 17 months), its effects on rice NUE and system-level N dynamics under different CA practices remained unexamined.

To address this gap — and the broader need for mechanistic, crop-specific, and context-sensitive assessments of legume effects on NUE in CA systems (Franke et al. 2018; Giller et al. 2015; Yao et al. 2025) — we re-analysed data from a three-year field trial in the Midwestern highlands of Madagascar. Our hypotheses were: (i) stylo integration enhances rice NUE by increasing N supply; (ii) the effect of stylo on NUE depends on soil and residue management; and (iii) rice cultivar traits interact with legume effects to influence physiological N utilization. To trace N dynamics, we applied three complementary methods: (i) the difference method, (ii) direct ^15^N labelling, and (iii) indirect ^15^N labelling (Hauck and Bremner 1976), which allowed us to distinguish N uptake from legumes, native soil, and applied fertilizers. The experimental design combined three fertilizer inputs (none, farmyard manure, mineral fertilizer), two cropping systems (with *vs.* without stylo), two soil management practices (tilled *vs.* no-till), and two residue management strategies (biomass removal *vs.* restitution).

## Material and methods

### Study site

The field experiment was located next to the village of Ivory (19°33′36.1″ S, 46°24′34.7″ E, 932 m asl), in the district of Betafo, region Vakinankaratra, Madagascar. The tropical savannah climate, described in detail by Zemek et al. (2018), is characterized by distinct dry and rainy seasons. Because the onset of the rains is erratic, the cropping season for upland rice starts between mid-November and mid-December and ends with the harvest of rice at the end of March. The cumulative rainfall received in rainy season 1 (2010–11), 2 (2011–12) and 3 (2012–13) was 1800, 980 and 1450 mm (Fig. A, supplementary material). The soil was classified as a haplic Ferralsol (WRB 2014) and has a sandy clay loam texture (49% sand, 25% silt and 26% clay) in the top soil layer (0–0.2 m) (Randriamanantsoa et al. 2013). It is acidic and has a low total N, phosphorus (P) and organic matter content (Table 1). The cropping history of the experimental site, which had been hitherto farmer-managed, was as follows: upland rice (2004–05), maize (2005–06), bambara groundnut (*Vigna subterranea*) intercropped with cassava (2006–07), two years of natural fallow (2007–09) and soybean (2009–10). Nutrient management from the 2006–07 to 2009–10 seasons did not include fertilizer application, and no reliable information was available for earlier seasons.

**Table 1:** Selected soil properties at the study site at Ivory measured in August 2010

Soil depth (m)		0–0.1	0.1–0.2	0.2–0.3
^a^Total C	g kg^−1^ soil	11.5 ± 0.2	10.2 ± 0.2	8.0 ± 0.1
^a^Total N	g kg^−1^ soil	0.9 ± 0.1	0.8 ± 0.2	0.6 ± 0.1
^b^Total P	mg kg^−1^ soil	424.0 ± 72.5	389.0 ± 72.1	340.2 ± 68.1
CEC	meq/100 g	1.5 ± 0.2	1.4 ± 0.1	1.3 ± 0.2
pH	(H_2_O)	4.9 ± 0.2	5.0 ± 0.2	5.0 ± 0.3
Bulk density	g cm^−3^	1.1 ± 0.1	1.2 ± 0.1	1.2 ± 0.1

Shown are averages ± standard deviation (*n* = 48)

^a^Total C and N analysed with NCS elemental analyser (Flash EA 1112 series, Thermo Electron Corporation, Waltham, USA)

^b^Total P was determined by ashing 0.2 g of organic material at 550 °C for 4 h, dissolving the ash in 2 mL of 15 M HNO_3_ and making the volume to 100 mL using ddH_2_O, followed by colorimetric measurement of Pi with the Malachite green method (Vanveldhoven and Mannaerts 1987)

### Experimental design

The field experiment followed a factorial design in a randomized complete block arrangement with four replicates per treatment, as originally established and described by Zemek et al. (2018). Main plots measured 6 × 10 m and were arranged in randomized complete blocks. Soil management (tillage *vs.* no-tillage) and mulch application (residue retention *vs.* removal) were assigned as main plot factors, while fertilizer type (mineral vs. organic) was applied at the subplot level. Prior to the rice phase, stylo was grown in all plots for 17 months and managed as a fallow, serving as the standard biological N input. To assess indigenous soil fertility and natural productivity in the absence of legume effects, an additional treatment with bare fallow was included as a baseline reference. This factorial structure enabled evaluation of main and interactive effects of soil management, residue handling, and nutrient inputs on rice N use efficiency and related performance parameters. The specific management combinations implemented within this framework, including their abbreviations, are detailed in the subsequent description of treatments.

### Crop rotations

**Fig. 1:**
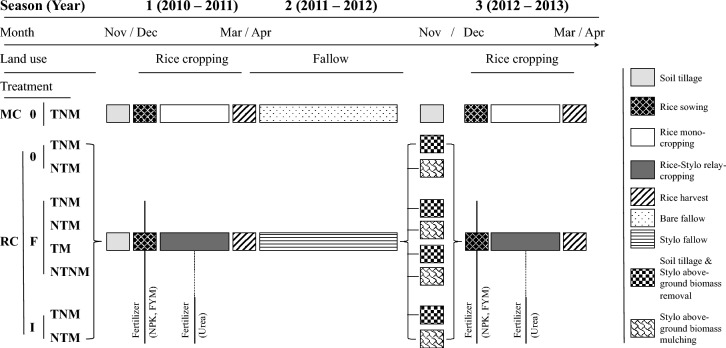
Timeline of experimental treatments. Treatments combined crop rotation (MC = rice mono-crop, RC = rice–stylo relay-crop), soil management (T = tilled, NT = no-till), mulch (NM = no mulch, M = stylo mulch), and fertilizer (0 = none, F = FYM, I = mineral fertilizer). Core treatments (RCTNM-0, RCNTM-0, RCTNM-F, RCNTM-F, RCNTM-I, RCTNM-I) and additional treatments (RCTM-F, RCNTNM-F, MCTNM-0) are shown. Rectangles indicate the different agricultural treatments (see legend). Fertilizer was applied at sowing, with a second application at panicle initiation for mineral fertilizer (vertical lines). Management practices common to all relay-crop treatments are summarized in a single line for clarity

The CA rotation followed a two-year cycle consisting of rice followed by stylo (*Stylosanthes guianensis*, cultivar CIAT 184) fallow. In the first cropping season (December 2010 – March 2011), upland rice (cultivar NERICA 4, WAB 450-I-B-P-91-HB) was relay-cropped with stylo. This was followed by a 17-month stylo fallow period (April 2011–October 2012). In the second cropping season (December 2012–March 2013), rice was again relay-cropped with stylo (Fig. 1). To assess indigenous soil fertility in the absence of legume effects or external inputs, a mono-cropped rice system was included as a control in both cropping seasons. These plots received neither fertilizer nor stylo and remained as bare fallow between rice crops (rice – bare fallow – rice).

### Crop, soil and stylo fallow biomass management

In the initial cropping season (2010–2011), all plots were tilled before sowing to ensure uniform soil conditions. Rice was relay-cropped with stylo and managed as described by Zemek et al. (2018). After rice harvest, stylo continued to grow as a fallow crop. During the fallow period (2011–2012), twice manual weeding was conducted at the onset of the rainy season to promote stylo establishment and canopy closure. Prior to the second cropping season (October 2012), stylo aboveground biomass was harvested at ground level. Average stylo shoot biomass across plots was 8.4 Mg DM ha^−1^ (± 1.8 SD). In plots without mulch application, all aboveground biomass, including surface litter, was removed. In mulch-designated plots, biomass was adjusted to a uniform level of 9 Mg DM ha^−1^ by adding or removing material as needed. Stylo shoots were chopped into 0.10–0.15 m pieces and evenly applied to the soil surface as mulch. Subsequent soil and crop management followed the respective treatment design, except that in the second cropping season, stylo regenerated from the soil seed bank and was not re-sown.

### Fertilizer management

According to treatment, rice received no fertilizer, farmyard manure (FYM), or mineral fertilizer. Farmyard manure (5 Mg DM ha^−1^) was derived from cattle (*Bos indicus*) and provided by local farmers. It consisted of a mixture of soil, plant residues (bedding and feed material), and manure, produced in overnight paddocks—a composition locally referred to as *poudrette de parc* (Rakotondravelo and Arsène 2011). Mineral fertilizer was applied as a compound NPK blend (33 kg N ha^−1^, 29 kg P ha^−1^, 40 kg K ha^−1^) at sowing, followed by a top dressing of 37 kg N ha^−1^ as urea at the panicle initiation stage. An overview of fertilizer types and associated N and P input rates applied in cropping seasons one and two are provided in Table 2.

**Table 2:** Fertilizer type, total DM rates and amounts of applied N and P to rice in the main plots

Cropping season		1 (2010–11)			2 (2012–13)		
		Thereof applied element N and P (g m^−2^)					
Fertilizer	Rate (kg ha^−1^)	^a^N	^b^P	^c^C:N	^a^N	^b^P	^c^C:N
NPK (11, 22, 16)	300	3.3	2.9	–	3.3	2.9	–
Urea (46% N)	80	3.7	–	0.4	3.7	–	0.4
FYM (DM)	5000	6.8	1.6	12	7.6	1.6	12
Stylo mulch (DM)	9000	–	–	–	6.9	1.3	57

^a^Total N analysed with NCS elemental analyser (Flash EA 1112 series, Thermo Electron Corporation, Waltham, USA)

^b^Total P was determined by ashing 0.2 g of organic material at 550 °C for 4 h, dissolving the ash in 2 mL of 15 M HNO3 and making the volume to 100 mL using ddH2O, followed by colorimetric measurement of Pi with the Malachite green method (Vanveldhoven and Mannaerts 1987)

^c^mass ratio

### Treatments

In the second cropping season, treatments combined different levels of crop rotation, soil and mulch management, and fertilizer input. The main factors were: (i) cropping system – rice mono-crop (MC) or rice–stylo relay crop (RC), (ii) soil management – tillage (T) or no-tillage (NT), (iii) mulch – no stylo mulch (NM) or stylo mulch (M), and (iv) fertilizer type – none (0), FYM (F), or mineral fertilizer (I). This factorial design resulted in a total of nine treatments (Fig. 1). Six core treatments included RCTNM-0, RCNTM-0, RCTNM-F, RCNTM-F, RCNTM-I, and RCTNM-I, where NT and M as well as T and NM were treated as combined factors (*i.e.*, NTM and TNM). To specifically isolate the effects of tillage and mulch, two additional RC treatments were introduced: RCTM-F (mulch incorporated by tillage) and RCNTNM-F (mulch removed, no tillage). The baseline treatment to test indigenous soil fertility with rice grown without fertilizer as mono-crop in tilled soil was named MCTNM-0.

### Assessment of rice N uptake from organic and mineral N inputs

Rice N uptake from stylo mulch, farmyard manure (FYM), and mineral fertilizer was quantified using both direct and indirect ^15^N labelling techniques (DLT and ILT, respectively) following Hood et al. (2008). The experiments were conducted in micro plots (1.8 × 1.6 m) nested within the main plots and demarcated in October 2011 using metal sheet frames inserted to a depth of 0.30 m (Fig. B, Supplementary Material). The properties of ^15^N-enriched materials and the respective N input rates applied in the DLT and ILT micro plots are provided in Table 3. A detailed description of labelling procedures for both fertilizer application and soil enrichment is available in the Supplementary Material (Fig. C).

**Table 3:** Amount and ^15^N enrichment of N applied with organic and mineral fertilizers to rice in micro plots using the direct (DLT) and indirect (ILT) labelling method in cropping season 2 (2012–13)

Method	Fertilizer	N	Mineral ^a^N	^15^N abundance	^b^C:N	Dose
		g N kg^−1^ DM	mg N kg^−1^ DM	atom%		g N m^−2^
DLT	^15^N labelled stylo mulch	7.6	78	0.68	57	6.9
	(^15^NH_4_)_2_SO_4_	21.0	224	11.60	–	3.3
	(^15^NH_2_)_2_CO	46.0	469	10.40	0.4	3.7
ILT	Stylo mulch	7.6	78	0.37	57	6.9
	FYM	13.5	737	0.37	14	6.7
	(NH_4_)_2_SO_4_	21.0	224	0.37	–	3.3
	(NH_2_)_2_CO	46.0	469	0.37	0.4	3.7

^a^0.01 M CaCl_2_ extractable mineral N (NH_4_^+^, NO_3_^−^)

^b^mass ratio

For the DLT, rice N uptake was assessed in three treatments: RCTNM-I and RCNTM-I using ^15^N-labelled mineral fertilizer, and RCNTM-0 using ^15^N-labelled stylo mulch. Rice was grown in the presence of ^15^N-labelled material, and uptake was determined by comparing ^15^N enrichment in plant tissue relative to the applied sources (see Eq. 1).

The ILT approach involved in situ ^15^N soil labelling during the preceding fallow season. Labelling was conducted in paired micro plots nested within each main treatment plot. Before rice sowing, ^15^N-labelled stylo biomass was harvested, sub-sampled, dried, chopped, and either removed (RCTNM-F, RCNTNM-F, RCTNM-I) or returned (RCNTM-F, RCTM-F, RCNTM-I) to the corresponding micro plot at a standardized rate of 9 Mg DM ha^−1^. Subsequently, one of the paired micro plots received non-labelled FYM or mineral fertilizer, while the paired control plot remained without fertilizer. In RCNTM-0, the ^15^N-labelled mulch was replaced with non-labelled mulch. Comparison of ^15^N enrichment in rice tissue between fertilizer-applied and no-fertilizer subplots enabled estimation of N uptake from the added fertilizer (see Eq. 3).

### Rice aboveground biomass sampling

At rice maturity, shoots were harvested at ground level. In each main plot, biomass was sampled from a 4 × 1.2 m area. In micro plots, all rice plants were harvested, but to minimize border effects, plants from the central rows were analysed separately from those in the two outer rows (Dourado-Neto et al. 2010). Rice grains (paddy rice) were manually separated from straw using a pedal-powered thresher. Subsamples of grain and straw were dried at 60 °C for 48 h to determine DM weight. Border-row samples from micro plots were excluded from further analysis (McDonagh et al. 1993). Grain yield was corrected to a standard moisture content of 0.14 g H_2_O g^−1^ (Yoshida et al. 1976).

### Rice root biomass sampling

Root DM was sampled at late flowering in core treatments (RCTNM-0, RCNTM-0, RCTNM-F, RCNTM-F, RCTNM-I, RCNTM-I). In each main plot, three sampling locations were selected randomly. At each location, four soil cores (Ø 0.08 m; to 0.30 m depth in 0.10 m increments) were taken in a rectangular arrangement: directly above a rice hill, and at half the inter-row, inter-line, and diagonal distances from the hill. This yielded 12 soil cores per depth and plot. Cores were soaked in water and roots were extracted through sequential sieving (5 mm, 2 mm, 0.5 mm mesh) to remove soil particles. Cleaned roots were placed in petri dishes and hand-sorted to remove weed roots and debris. Dry matter was determined after oven drying at 60 °C to constant weight. For each depth, the 12 samples per plot were pooled before further analysis.

#### Rice and stylo biomass sample preparation and analyses

Representative subsamples of stylo mulch, FYM, and rice DM (grain, straw, roots) were shipped to Switzerland for laboratory analysis. Samples were milled to a final particle size of 0.2 mm using a micro hammer mill (Culatti®, Switzerland) and an ultra-centrifugal mill (Retsch® ZM 200, Germany). For samples from main plots, total N and C contents were determined using an elemental analyser (Flash EA 1112, Thermo Electron Corp., USA) at the ETH Zurich research station in Eschikon. Micro plot samples of rice grain and straw were analysed for total N concentration and ^15^N isotopic enrichment using an isotope ratio mass spectrometer (Europa Tracer/20, Europa Scientific, UK) at the Stable Isotope Facilities, Department of Soil Science, University of Saskatchewan, Canada.

#### Calculating fertilizer N uptake using the DLT and ILT

For the DLT, the proportion of N in the rice plant that is derived from the applied labelled fertilizer expressed as Ndff (%) was calculated according to Hauck and Bremner (1976):

1 $$Ndff (\%)= \frac{{AE}_{+N}}{{AE}_{F}} x 100$$

where AE_+N_ is the atom% ^15^N excess of N in the rice plant that has received the labelled fertilizer and AE_F_ is the atom% ^15^N excess of N in the applied fertilizer. Atom% ^15^N excess denotes the ^15^N abundance of the rice plant minus the natural abundance of a reference rice plant grown under the same experimental conditions but without the application of ^15^N. The AE_F_ is derived by the difference of its ^15^N abundance and the atmospheric abundance of 0.3663 atom% ^15^N. To account for potential plant internal fractionation of ^15^N between different plant parts when determining the AE for rice shoots, which includes grain and straw, a weighted AE was calculated according to Danso et al. (1993):

2 $$weighted\,AE =\frac{\sum_{i = 1}^{n}{AE}_{i} \, x total\, N{\mathrm{i}}}{\sum_{i=1}^{n}total\, N{\mathrm{i}}}$$

where *i* is a given plant component and *n* the total number of plant components. Using the same principle of Eq. 2, a weighted AE was calculated for the two ^15^N labelled mineral fertilizers urea and (NH_4_)_2_SO_4_, which differed in their ^15^N abundance (Table 3).

For the ILT, the Ndff (%) derived from the applied non-labelled fertilizer was calculated according to McAuliffe et al. (1958);

3 $$Ndff (\%)= (1-\frac{A{E}_{+N}}{{AE}_{0N}} ) x 100$$

where AE_+N_ is the atom% ^15^N excess of rice plants grown on ^15^N labelled soil that has received non-labelled fertilizer and AE_0N_ is the atom% ^15^N excess of rice plants grown on ^15^N labelled soil without the addition of fertilizer.

For the DLT and ILT, the amount of fertilizer N (g N m^−2^) taken up in rice shoots (grain + straw) derived from the applied fertilizer (Ndff) was calculated as

4 $$Ndff= \frac{Ndff (\%)}{100}x TNU$$

where TNU refers to the total amount of N (g N m^−2^) taken up in the rice shoot (grain + straw) at harvest, which is determined by the N concentration (g N kg^−1^ DM) in the shoot components multiplied by the respective DM (kg DM m^−2^).

The percentage of fertilizer N (FNRec) recovered in the rice plant for the DLT and the ILT was calculated according to Hauck and Bremner (1976);

5 $$FNRec (\%)= \frac{Ndff}{{N}_{F}} x 100$$

where N_F_ is the amount in g N m^−2^ of applied fertilizer N.

The amount of N derived from the soil (Ndfs) in g N m^−2^ was calculated by the difference between TNU and the Ndff:

6 $$Ndfs= TNU - Ndff$$

#### Calculating fertilizer N recovery using the difference method

The apparent N recovery from mineral fertilizer and FYM by rice (ANRec%) was calculated using the difference method after Munoz et al. (2004).

7 $$ANRec \left(\%\right)= \frac{\left(Nuptake\, in\, amended\, rice-Nuptake in nonamended \,rice\right)*100}{N\, applied }$$

where N uptake in amended rice is N expressed in g N m^−2^ taken up by grain, straw or both in rice that has received mineral fertilizer or FYM, while N uptake in no-fertilizer rice is the N taken up in the same organs grown in the absence of mineral fertilizer or FYM.

The apparent N recovery from the stylo mulch was calculated as follow:

8 $$ANRec \left(\%\right)= \frac{\left(Nuptake\, in \,mulched \, rice-Nuptake\, in\, nonmulched\, rice\right)*100}{Napplied, with\, mulch }$$

where N uptake in mulched rice is the sum of N taken by grain, straw or both while N uptake in non-mulched rice is the N taken up in the absence of mulching.

#### Determination of rice physiological N utilization efficiency

The physiological N utilization efficiency in g DM g^−1^ N (pNUE, g DM g^−1^ N) was calculated after Ladha et al. (2005):

9 $$pNUE = \frac{GY}{TNU}$$

where GY indicates grain yield (g DM m^−2^) and TNU refers to the total amount of N (g N m^−2^) taken up in the rice plant shoot (grain + straw) at harvest.

#### Statistical analyses

All statistical analyses were performed using R version 4.3.2 (R-Core-Team 2023). For core treatments (RCTNM-0, RCNTM-0, RCTNM-F, RCNTM-F, RCTNM-I, RCNTM-I), the effects of fertilizer and combined soil–residue management (tillage without mulch *vs.* no-tillage with mulch), and their interaction, were tested using two-way analysis of variance (ANOVA). To separate the effects of soil *vs.* mulch management more specifically, FYM-amended treatments (RCTNM-F, RCTM-F, RCNTM-F, RCNTNM-F) were also analysed via two-way ANOVA. In all models, block effects were included as a random factor. Post-hoc comparisons were conducted using Fisher’s least significant difference (LSD) test at *α* = 0.05, with Bonferroni correction for multiple comparisons. Standard errors of difference (SED) were derived from the respective ANOVA output. Welch’s two-sample t-test was applied to assess: (i) differences between the MC control (MCTNM-0) and the mean of no-fertilizer RC treatments (RCTNM-0, RCNTM-0), and (ii) seasonal differences in N uptake and dry matter parameters. Normality and homoscedasticity were verified using the Shapiro–Wilk and Levene’s tests, respectively. If assumptions were violated, robust hypothesis testing based on 20% trimmed means was performed using the WRS package (Wilcox 2012).

## Results

### Rice grain yield

**Fig. 2:**
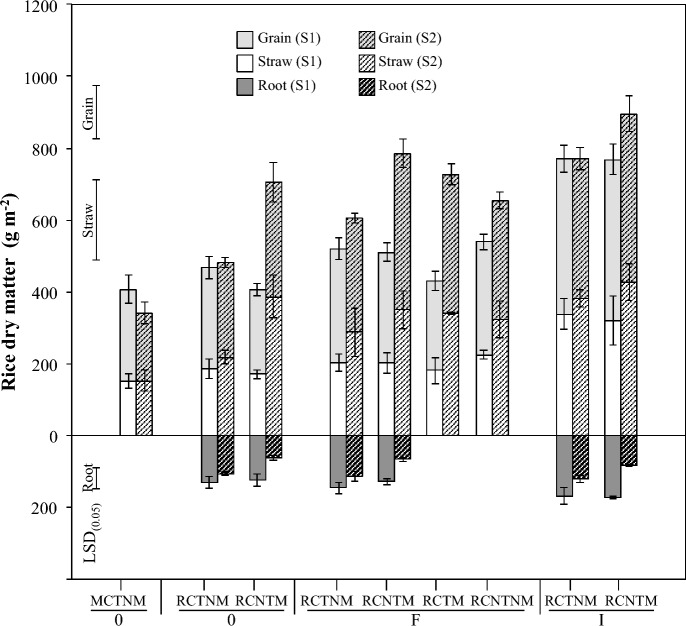
Rice DM production prior and after (dotted bars) stylo fallow in main plots of the field trial installed at the study site (Ivory, Madagascar). Root DM (0–0.3 m soil depth) (not determined in all treatments). Mono-cropping = MC, Relay-cropping = RC; Tillage = T, No-tillage = NT, Stylo mulch = M, No stylo mulch = NM; no fertilizer = 0, FYM = F and NPK + urea = I. Error bars show ± 1 SE (*n* = 4). *LSD *_*(0.05)*_ for DM of grain (147 g m^−2^), straw (224 g m^−2^) and root (59 g m.^−2^)

While crop rotation with stylo did not significantly increase rice grain yield overall, a positive trend was observed after stylo fallow, with yields averaging 366 g DM m^−2^ compared to 337 g DM m^−2^ before the fallow (Fig. 2; Table A supplementary Material). After the fallow period, grain yield in RC treatments (RCTNM-0, RCNTM-0) was 53% higher than in the MC control (MCTNM-0; *p* < 0.05). Across seasons, mineral fertilizer increased grain yield by up to 71%, and FYM by 30%, relative to no-fertilizer controls. However, the FYM effect was not statistically significant. After stylo fallow, no-till with mulch (NTM) treatments yielded 26% more grain than tilled, non-mulched (TNM) treatments (*p* < 0.01). Separating soil and mulch effects in FYM-amended plots revealed 26% higher yields with stylo mulch than without (*p* < 0.05; Table B supplementary Material).

### Rice straw biomass

Straw production of RC rice increased by 44% after stylo fallow (342 *vs*. 237 g DM m^−2^; *p* < 0.001). In the MCTNM-0 control, straw biomass remained stable across both seasons (151 vs. 153 g DM m^−2^). Fertilizer effects followed the trend: NPK + urea > FYM ≥ none (Fig. 2). In the second season, straw biomass was 31% higher in NTM compared to TNM treatments (*p* < 0.05), although mulch and tillage effects within FYM treatments were not statistically significant.

### Rice root biomass

Rice root DM in the top 0.30 m ranged from 61 to 172 g DM m^−2^. Across treatments and seasons, 70%, 18%, and 12% of root biomass were located in the 0–0.1 m, 0.1–0.2 m, and 0.2–0.3 m layers, respectively (Fig. 2). Overall, root biomass was 36% lower than before the stylo fallow (*p* < 0.001). Across seasons, mineral fertilizer increased root DM by 22–32% (*p* < 0.05), while FYM had only minor effects. In the second season, root biomass in NTM treatments was 20% lower than in TNM treatments (*p* < 0.05).

### Rice grain N concentration

Overall, rice grain N concentration was 20% higher before the stylo rotation than after (*p* < 0.05), whereas straw N concentration remained stable and root N concentration increased by 30% (*p* < 0.05; Table 4). In the initial cropping season, grain N concentration ranged narrowly between 14 and 15 g N kg^−1^ DM across treatments. Following stylo fallow, fertilizer effects became more pronounced (*p* < 0.001), with grain N concentrations following the order: NPK + urea > no fertilizer ≥ FYM. A significant interaction between soil and mulch management was observed in FYM-amended plots (*p* < 0.01; Table 5): under no-till conditions, stylo mulch had little effect, but under tilled conditions, grain N concentration was on average 26% higher when mulch was applied.

**Table 4:** Rice grain (GNC), straw (SNC) root (RNC) N concentration and physiological utilization efficiency (pNUE) in core treatments (RCTNM-0, RCNTM-0, RCTNM-F, RCNTM-F, RCNTM-I and RCTNM-I) and the mono-crop treatment (MCTNM-0) prior and after the fallow season

Cropping season		1 (2010–11)				2 (2012–13)			
Plot		Main				Main			
^a^Management		GNC	SNC	^b^RNC	pNUE	GNC	SNC	^b^RNC	^c^pNUE
		g N kg^−1^ DM			g DM g^−1^ N	g N kg^−1^ DM			g DM g^−1^ N
Fertilizer (FM)									
0		13.9	5.5	8.3	57	11.4*	5.0	13.3*	61
F		14.7	4.8	8.4	56	10.6*	4.1	12.2*	72*
I		14.6	6.7	8.9	51	14.2	5.3*	13.5*	52
*SED*		*0.54*	*0.25*	*0.45*	*1.7*	*0.42*	*0.40*	*0.56*	*3.8*
*LSD*_*0.05*_		*1.45*	*0.66*	*1.21*	*4.7*	*1.14*	*1.08*	*1.51*	*10.2*
^d^Soil + stylo mulch (SRM)									
TNM		14.5	5.7	8.7	54	12.0*	4.8	13.9*	63
NTM		14.3	5.6	8.5	55	12.2*	4.8	12.1*	60
*SED*		*0.44*	*0.20*	*0.37*	*1.4*	*0.34*	*0.33*	*0.46*	*3.1*
*LSD*_*0.05*_		*0.94*	*0.43*	*0.78*	*3.0*	*0.73*	*0.70*	*0.97*	*6.6*
Overall mean		*14.4*	*5.7*	*8.6*	*55*	*12.1*	*4.8*	*13.0*	*62*
^e^MCTNM-0		14.2 (0.45)	5.2 (0.13)	*nd*	58 (1.6)	12.2* (0.35)	6.4 (0.13)	*nd*	58 (3.7)
*t – test (p)*		*ns*	*ns*	*na*	*ns*	*ns*	*ns*	*na*	*ns*
*Source of variation*	df								
Block	3	*ns*	*ns*	**	*ns*	*ns*	*ns*	*ns*	*ns*
FM	2	*ns*	***	*ns*	*	***	*	*ns*	*
SRM	1	*ns*	*ns*	*ns*	*ns*	*ns*	*ns*	*	*ns*
FM x SRM	2	*ns*	*ns*	*ns*	*ns*	*ns*	ns	ns	*ns*

Means, *n* = 8 (FM), *n* = 12 (SRM); Not significant (*ns*); * significant at the 0.05 level, ** significant at the 0.01 level, *** significant at the 0.001 level*;* standard error of difference (SED); Not applicable (*na*)*,* Not determined (*nd*); Asterix* in rows indicate significant (t-test, *p* < 0.05) differences for the respective characteristics between cropping seasons

^a^Tillage + No-Mulch = TNM, NTM = No-Tillage + Mulch; no fertilizer = 0 and FYM = F and NPK + urea = I

^b^Root DM (0–0.3 m soil depth)

^c^GY / TNU

^d^Soils in all plots were tilled in cropping season 1. Statistical analysis was conducted to test for initial treatment differences, irrespective of SRM

^e^Results for the MCTNM-0 treatment were not included in the ANOVA. Shown are the mean (± standard deviation) and the t-test probabilities of significant difference to the mean of the RC-0 treatments (RCTNM-0, RCNTM-0)

**Table 5:** Rice grain (GNC), straw (SNC) N concentration and physiological N utilization efficiency (pNUE) in FYM-amended treatments (RCTNM-F, RCNTM-F, RCTM-F, RCNTNM-F) after the fallow season

Cropping season		2(2012–13)		
Plot		Main		
^a^Management		GNC	SNC	^b^pNUE
		gNkg^−1^DM		G DM g^−1^N
Soil (SM)				
T		11.3	3.8	69
NT		10.9	4.2	69
Stylo mulch (RM)				
NM		10.2	3.8	73
M		11.9	4.2	65
*SED*		0.28	0.38	3.1
*LSD*_*0.05*_		0.64	0.86	7.0
*Overall mean*		11.1	4.0	69
*Source of variation*	*df*			
Block	3	**	ns	**
SM	1	ns	ns	ns
RM	1	**	ns	ns
SM × RM	1	**	ns	ns

Means, *n* = 8 (SM), *n* = 8 (RM); Not significant (*ns*); * significant at the 0.05 level, ** significant at the 0.01 level, *** significant at the 0.001 level*;* standard error of difference (SED)

^a^Tillage = T, No tillage = NT; No stylo mulch = NM, Stylo mulch = M

^b^GY / TNU

### Rice straw N concentration

Fertilizer type was the only factor significantly affecting straw N concentration (*p* < 0.001). In both cropping seasons, straw N levels were highest with mineral fertilizer (5–7 g N kg^−1^ DM), followed by no-fertilizer (5–6 g) and FYM-amended (4–5 g) treatments (Table 4).

### Root N concentration

Root N concentration averaged 9 g N kg^−1^ DM in the first, and 13 g in the second cropping season. In the second season, both soil and mulch management significantly influenced root N levels (*p* < 0.05), with concentrations 15% higher in TNM compared to NTM treatments (Table 4).

### Rice N uptake partitioning

**Fig. 3:**
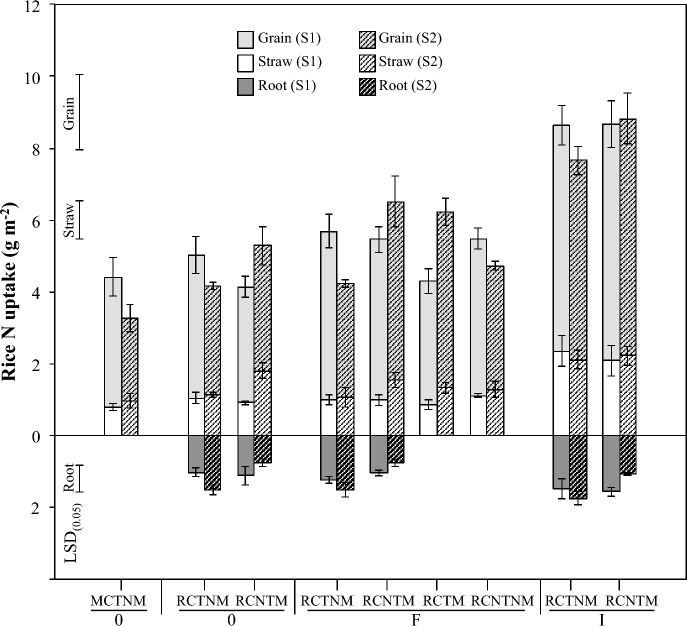
Rice N uptake prior and after (dotted bars) the fallow season in main plots. Root N (DM from 0–0.3 m soil depth). Mono-cropping = MC, Relay-cropping = RC; Tillage = T, No-tillage = NT, Stylo mulch = M, No stylo mulch = NM; no fertilizer = 0, FYM = F and NPK + urea = I. Error bars on bars show ± 1 SE (*n* = 4). *LSD *_*(0.05)*_ for grain (2.1 g m^−2^), straw (1.1 g m^−2^) and root (0.7 g m^−2^) N uptake

Across all treatments and seasons, the partitioning of N uptake in rice followed a consistent ratio of approximately 3:1:1 among grain, straw, and root compartments, respectively (Fig. 3; Table C supplementary Material).

### Rice grain N uptake

Total N uptake in rice grain ranged from 2.3 to 6.4 g N m^−2^. Crop rotation with stylo had no significant effect on grain N uptake. Fertilizer application significantly increased grain N uptake in both seasons (*p* < 0.001), following the pattern: NPK + urea > FYM ≥ no fertilizer. Rice with mineral fertilizer absorbed nearly twice as much N as no-fertilizer rice. Soil and mulch management also influenced grain N uptake (*p* < 0.05), with NTM treatments outperforming TNM by 28%. In FYM-amended treatments, grain N uptake was 48% higher with stylo mulch than without (*p* < 0.05; Table B supplementary Material).

### Rice straw N uptake

Straw N uptake ranged from 0.8 to 3.6 g N m^−2^. No significant effect of the fallow season was detected. However, fertilizer significantly increased straw N uptake in both seasons (*p* < 0.01 and *p* < 0.05), with mineral fertilizer resulting in 50–120% higher uptake compared to FYM and no-fertilizer treatments. Straw N uptake was also 35% higher in NTM than TNM plots (*p* < 0.05), although separating soil from mulch effects revealed no significant differences within FYM treatments.

#### Rice root N uptake

Root N uptake ranged from 1 to 2 g N m^−2^ and remained stable across cropping seasons. Fertilizer effects followed the trend: NPK + urea ≥ FYM = none, though statistical significance was limited to the first season. After stylo fallow, soil and mulch management had a strong effect (*p* < 0.001), with TNM plots showing 78% higher root N uptake compared to NTM.

#### Nitrogen derived from fertilizer in rice shoots

**Fig. 4:**
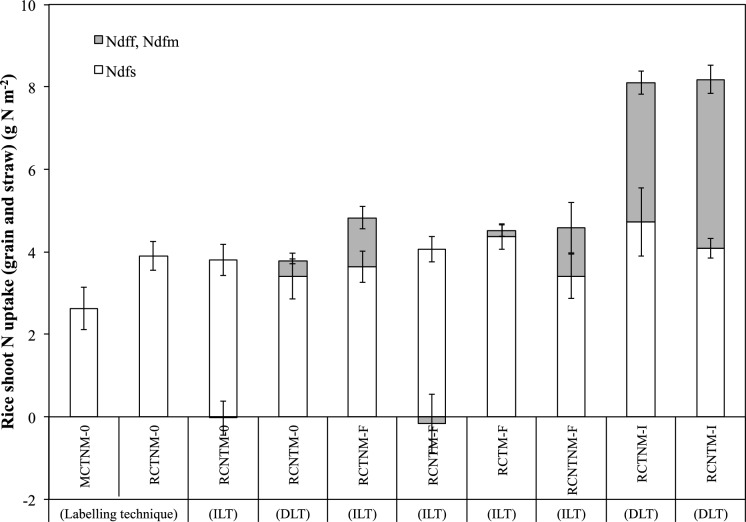
Rice shoot (grain and straw) N uptake derived from the soil (Ndfs) and from fertilizer (Ndff) or stylo mulch (Ndfm) in micro plots determined by the direct (DLT) and indirect (ILT).^15^N labelling method in the field trial. Mono-cropping = MC, Relay-cropping = RC; Tillage = T, No-tillage = NT, Stylo mulch = M, No stylo mulch = NM; no fertilizer = 0, FYM = F and NPK + urea = I. Error bars on bars show ± 1 SE (*n* = 4)

Across treatments and labelling methods, the proportion of N in rice shoots derived from external inputs (Ndff) followed the order: NPK + urea > FYM > stylo mulch (Fig. 4). Most Ndff values obtained via the ILT were consistent with those from the difference method, with a few exceptions. In three treatments—RCNTM-0 (stylo mulch), RCNTM-F, and RCTM-F (FYM)—the ILT yielded either negative or unrealistically low Ndff estimates. Using the DLT, Ndff from stylo mulch was 10% in the RCNTM-0 treatment. For FYM, average Ndff was 24% in both RCTNM-F and RCNTNM-F treatments, with no significant effect of soil management. For mineral fertilizer, Ndff values were 50% and 43% in RCNTM-I and RCTNM-I, respectively, without significant differences between treatments.

#### Nitrogen derived from soil in rice shoots

Nitrogen derived from soil (Ndfs) values in rice shoots were 35–73% higher in RC treatments with stylo mulch, FYM, or mineral fertilizer compared to the MC control (MCTNM-0; Fig. 4). Mineral fertilizer significantly increased soil-derived N uptake relative to MCTNM-0 (*p* < 0.001). Comparing FYM and mineral fertilizer treatments showed a tendency for higher Ndfs in TNM than in NTM systems (+ 7–13%), but differences were not statistically significant.

#### Fertilizer N recovery in rice

Across treatments, fertilizer N recovery (FNRec%) in rice shoots followed the order: NPK + urea (52%) > FYM (18%) ≥ stylo mulch (5%), as determined by both the DLT and ILT (Table D, Supplementary Material). In all cases, FNRec% was consistently higher in grain than in straw fractions. No significant effects of soil management were observed on FNRec% from FYM. Likewise, FNRec% values for mineral fertilizer did not differ significantly between the DLT and ILT. Estimates of apparent N recovery (ANRec%) from the difference method closely matched FNRec% values from ^15^N labelling for mineral fertilizer and FYM. However, ANRec% of stylo mulch could not be determined using this method.

#### Physiological N use efficiency in rice

Physiological N use efficiency (pNUE), defined as grain DM produced per unit of N taken up, ranged from 51 to 73 g DM g^−1^ N across RC rice treatments (Tables 4 and 5). In the MC control (MCTNM-0), pNUE was stable across cropping seasons, averaging 58 g DM g^−1^ N. Fertilizer type significantly influenced pNUE (*p* < 0.05), with FYM treatments consistently yielding values equal to or higher than those observed under NPK + urea. Following stylo fallow, pNUE in FYM-amended plots increased by 29% compared to values before the fallow (*p* < 0.05). Separating mulch from soil management within FYM treatments revealed a trend toward lower pNUE with mulch (− 11%), while soil tillage had no detectable effect.

## Discussion

### Effect of crop rotation with stylo

Crop rotation with stylo maintained soil N supply under low-input conditions, as indicated by stable rice yields and increased straw production (Fig. 2). This occurred even without fertilizer and aligns with previous studies using stylo as a fallow crop (Becker and Johnson 1998; Dusserre et al. 2020; Saito et al. 2006), supporting Hypothesis (i) that stylo integration can enhance rice NUE via increased N supply. Enhanced performance of relay-cropped rice likely reflects higher soil N availability, consistent with increased mineral N concentrations and N uptake compared to mono-cropped systems (Zemek et al. 2018).

Although these effects cannot be attributed solely to N mineralization from stylo residues, increased straw biomass suggests broader stimulation of shoot growth, likely driven by improved N availability (Blume et al. 2016). Beyond N benefits, legume rotations may also enhance P availability, suppress soil-borne pests, and improve soil structure and microbial activity (Franke et al. 2018), illustrating how legume integration can boost both productivity and resilience in smallholder CA systems.

### Effect of post-fallow soil cover with stylo mulch

Assessing stylo mulch as a post-fallow ground cover revealed three key insights for N availability and rice uptake:

- Limited contribution of mulch-derived N – Mulch contributed only a small fraction of total N in rice shoots in no-fertilizer, no-till plots, consistent with typical legume residue recovery (10–30%; Crews and Peoples 2005). Limited recovery is likely due to its high C:N ratio (55:1) and partial decomposition (61% by harvest; Zemek et al. 2018).
- No reduction in fertilizer N recovery – Contrary to concerns about microbial N immobilization, mulch did not impair mineral fertilizer N uptake. Fertilizer N recovery averaged 52%, above the global benchmark of 44% (Ladha et al. 2005), likely because surface-applied residues decompose more slowly and compete less for N than incorporated residues (Coppens et al. 2007).
- Improved microclimate and soil N release – Mulch increased soil moisture by 11–16% at 5 cm depth (Zemek 2016), promoting microbial activity and organic matter turnover (Blair et al. 1994), enhancing mineral N release. This supports Hypothesis (ii) that stylo effects on rice NUE depend on soil and residue management.

Fertilizer N recovery was higher in mulched plots (59% vs. 48% without mulch via DLT), although ILT-based estimates showed the opposite trend (56% vs. 42%), reflecting methodological sensitivity (Table D, Supplementary Material). These results suggest that mulch enhances N retention and reduces losses.

For smallholder farmers, targeted mulch application can increase fertilizer cost-efficiency. Ndff values further show mulch–fertilizer interactions: FYM-derived N accounted for 24% of rice shoot N, mineral fertilizer 50% vs. 43% in RCNTM-I and RCTNM-I, and stylo mulch 10% in RCNTM-0 (DLT), indicating that mulch and soil management influence both total N uptake and the proportional contribution from different N sources.

### Effect of post-fallow soil management

Tillage had limited effects on rice N uptake, though root biomass was affected. Fertilizer N recovery from FYM averaged 18%, similar to temperate ruminant manure systems (Bosshard et al. 2009; Munoz et al. 2004). Grain N uptake differed only slightly between tilled (RCTNM-F) and no-till (RCNTNM-F) plots (+ 7%), with no statistical significance. While tillage can enhance organic matter mineralization and N availability (Corbeels et al. 2006), our results align with a meta-analysis across 16 African countries showing no consistent yield advantage for conventional tillage over reduced or no-till (Corbeels et al. 2020).

Root biomass in the top 0–0.3 m declined by 37% after stylo fallow, slightly more under no-till, consistent with previous studies in Madagascar (Dusserre et al. 2012). This pattern likely reflects higher surface bulk density in no-till plots. A significant difference in bulk density, however, was observed only in the top 0–0.1 m post-harvest in RCTM-F (1.01 g cm⁻^3^ vs. 1.09 g cm⁻^3^ across other treatments; Zemek 2016). Climatic conditions—post-fallow rainfall 20% below average with higher potential evapotranspiration (Fig. D, Supplementary Material)—may also have promoted deeper root allocation. NERICA 4 responds to drought by increasing rooting below 0.3 m (Blume et al. 2016; Menge et al. 2016).

Beyond individual tillage effects, mulch–tillage interactions influenced N dynamics. Incorporating stylo mulch into tilled soils increased grain yield (388 g DM m⁻^2^) and N uptake (4.9 g N m⁻^2^) compared to non-mulched tilled systems (331 g DM m⁻^2^; 3.4 g N m⁻^2^; Figs. 2, 3). This likely reflects accelerated residue decomposition, enhanced microbial activity, and improved aeration, emphasizing that mulch benefits depend on both chemical composition and physical management.

### Effect of fertilizer addition

Rice shoot biomass and N uptake were primarily driven by fertilizer inputs. The highest shoot N uptake (8–9 g N m⁻^2^) and grain yields (429–449 g DM m⁻^2^) occurred under mineral fertilizer, reaching the upper range reported for upland rice among local family farmers (~ 440 g DM m⁻^2^; Bruelle et al. 2015). This reflects synchronized nutrient supply during critical growth stages, particularly panicle initiation, and the immediate availability of N, P, and K (Fageria 2007). Fertilizer N recovery averaged 52%, exceeding global benchmarks for upland rice (Lee 2021).

Mineral fertilizer also increased soil-derived N (Ndfs), suggesting a priming effect on native soil N (Kuzyakov et al. 2000). Compared to MCTNM-0, Ndfs was significantly higher in mineral-fertilized treatments (p < 0.001). FYM-treated plots showed a moderate increase in Ndfs under tillage (+ 7–13%), though not statistically significant. These findings highlight the importance of accounting for both direct and indirect fertilizer contributions when evaluating NUE in CA systems.

FYM improved grain yields by 20–30% relative to no-fertilizer RC treatments but remained less effective than mineral fertilizer, likely due to slower N mineralization and lower P supply (Bosshard et al. 2011; Rufino et al. 2006). Delayed nutrient release can lead to temporal asynchrony with crop demand.

Fertilizer inputs also affected physiological NUE (pNUE, grain DM per unit N uptake). Mineral-fertilized rice averaged 52 g DM g⁻^1^ N, below the optimum of 68 g DM g⁻^1^ N for modern varieties (Dobermann and Fairhurst 2000), suggesting genotypic or co-limiting nutrient constraints. In contrast, FYM-treated rice showed a marked increase in pNUE after the fallow (72 *vs.* 56 g DM g⁻^1^ N), with higher straw biomass and modest yield gains, while grain N concentration declined (14.7 → 10.6 g N kg⁻^1^ DM). This pattern reflects a dilution effect: enhanced biomass under adequate P supply is not matched by proportional grain N accumulation, as observed in lowland rice (Saleque et al. 2001). Stylo residues combined with FYM likely improved P availability, allowing more efficient use of absorbed N for biomass. However, reduced grain N indicates persistent N limitation during grain filling, showing that higher pNUE reflects nutrient imbalances rather than a true improvement in physiological efficiency—consistent with Hypothesis (iii) that cultivar traits interact with legume effects to influence physiological N utilization.

These findings underscore the importance of nutrient interactions when designing fertilizer strategies for CA systems. Aligning nutrient release with crop demand, integrating organic and mineral inputs, and considering cultivar responses are key to enhancing NUE and system sustainability, with practical relevance for smallholder farmers.

### Fertilizer interactions and biomass productivity

High N recovery under mineral fertilizer, combined with the moisture-conserving effects of stylo mulch, highlights positive interactions between organic residue management and mineral inputs in CA systems. Similar synergies have been reported where surface-applied organic materials improve nutrient retention and uptake efficiency when used with mineral fertilizers (Gentile et al. 2009). Integrated nutrient management has been shown to enhance NUE through synchronized nutrient release and complementary resource use (Grahmann et al. 2014).

In this study, stylo mulch maintained favourable soil moisture throughout the growing season, likely supporting root function and nutrient acquisition during critical growth phases. This contributes to the high fertilizer N recovery (> 50%) observed in mineral-fertilized plots. FYM supplied additional N and P but released nutrients more slowly, underscoring the importance of aligning nutrient availability with crop demand to maximize efficiency.

Overall, these results reinforce the principles of integrated soil fertility management (ISFM), which advocate combining organic and mineral inputs to improve soil fertility, crop performance, and resilience in low-input systems (Vanlauwe et al. 2010). For smallholder rice-based CA systems, such strategies can enhance sustainability, resource-use efficiency, and economic returns, offering tangible benefits for farmers managing nutrient-limited soils.

### Implications for smallholder farmers

This study has several practical implications for smallholder farmers in sub-Saharan Africa. Legume rotation with stylo can sustain or increase rice yields without continuous fertilizer use, reducing dependence on costly external inputs and improving economic resilience. This supports broader evidence that legume integration in CA systems stabilizes nutrient balances and maintains productivity under low-input conditions (Kermah et al. 2018).

Increased straw biomass offers additional socioeconomic benefits. Rice straw serves multiple purposes—livestock feed, bedding, thatching, or brick making—providing farmers with economic incentives to adopt legume-based CA practices (Reddy et al. 2003). By enhancing both grain and co-product outputs, stylo integration promotes farm-level circularity and diversified income opportunities.

The lack of significant differences in N uptake between tilled and no-till plots suggests that simplified, low-labour CA practices, such as direct seeding or no-till, can achieve similar NUE. This reduces labour and energy requirements, making CA adoption more accessible for resource-constrained farmers.

Overall, legume-based CA systems improve yield stability and resilience under variable climatic conditions (van Vugt et al. 2018). The combined agronomic, economic, and ecological benefits of stylo integration offer a practical pathway toward sustainable intensification, better soil health, and enhanced adaptive capacity for smallholder communities.

## Conclusion

This study builds on Zemek et al. (2018) and provides new insights into the short-term effects of integrating stylo into CA systems. It delivers mechanistic, crop-specific, context-sensitive evidence of legume effects on NUE. Our results confirm the study hypotheses: stylo integration can enhance rice NUE by increasing N supply; its effects depend on soil and residue management; and cultivar traits interact with legume inputs to influence physiological N utilization. Upland rice yields were maintained—or even improved—during the first year of CA, regardless of fertilizer input. Stylo mulch contributed modestly to direct N uptake but improved soil moisture and microclimatic conditions, enhancing N mineralization and availability. In contrast, root and litter residues may have contributed more readily available N, suggesting a potentially greater role in soil N dynamics that warrants further investigation. Fertilizer remained the main driver of yield and N uptake, with mineral fertilizer increasing grain yield by up to 71% over the no-fertilizer control. Reduced grain N concentrations in no-fertilizer treatments raise nutritional concerns for rice-dependent populations in regions like Madagascar. These findings underscore the importance of balanced nutrient supply and targeted management to improve NUE in smallholder CA systems—not only through greater uptake but also via more efficient conversion into harvestable yield. This study demonstrates that legume integration, combined with organic–mineral inputs, can improve NUE through biological and management mechanisms, though trade-offs exist: while stylo biomass is valuable as livestock fodder, its removal reduces soil N inputs. Future research should explore partial biomass harvesting and manure recycling to maintain nutrient balances and support integrated nutrient cycling in low-input CA systems.

## Supplementary Information

Below is the link to the electronic supplementary material.

Supplementary file1 (DOCX 611 KB)

## Data Availability

Datasets analyzed during the current study are available from the corresponding author on reasonable request.

## Abbreviations

ANRec: Apparent N recovery from using the difference method
BNF: Biological N fixation
CA: Conservation agriculture
DLT: Direct labelling technique
DM: Dry Matter
FNRec: Fertilizer N recovered in the rice plant using DLT or ILT
FYM: FarmYard manure
ILT: Indirect labelling technique
MC: Mono-cropping
N: Nitrogen
Ndff: Amount of N derived from the applied fertilizer
Ndfs: Amount of N derived from the soil
NUE: N use efficiency
P: Phosphorus
pNUE: Physiological N use efficiency
RC: Relay-cropped
