# Supplementary material for: Nitrogen use efficiency and yield gains from Stylosanthes guianensis integration in upland rice: insights from a 15N-labelling study under conservation agriculture
Source: Nutr Cycl Agroecosyst. 2025 Dec 8;132(1):5. doi: 10.1007/s10705-025-10458-w (PMC12685974; doi:10.1007/s10705-025-10458-w)
Supplement: Supplementary file 1 — Supplementary file1 (DOCX 611 KB) [file 10705_2025_10458_MOESM1_ESM.docx]

**Supplementary material**

|  |
| --- |
| **Fig. A** Monthly rainfall received at the study site (Ivory, Madagascar) in rainy season 1 (2010-11), 2 (2011-12) and 3 (2012-13) and the 9-year average (2004-2013) |

|  |
| --- |
| **Fig. B** Schematic overview of the direct and indirect labelling technique to assess N uptake by rice from mineral fertilizer (I), from FYM (F) and from stylo mulch (M) in micro plots installed in main treatment plots (main plot size 10 m x 6 m, micro plot size 1.6 m x 1.8 m) at the study site). Control micro plots (C) required for the indirect labelling approach did not receive any fertilizer |

**Description of ^15^N soil labelling and ^15^N fertilizer application**

*^15^N fertilizer application – DLT*

For rice N uptake from mineral fertilizer, ^15^N-enriched ammonium sulphate was applied at sowing, and ^15^N-enriched urea at panicle initiation. Additionally, 29 kg P and 40 kg K ha^-^¹ (as KH_2_PO_4_ and KCl) were applied to match the amounts in the compound NPK fertilizer used in the main plots. Nutrient salts were dissolved in tap water and applied homogeneously at 1 L m^-^² using a watering can. At sowing, stylo mulch was temporarily removed (if present) to ensure direct soil application. At panicle initiation, the dissolved urea was poured onto the remaining stylo mulch, followed by additional tap water to wash any labelled urea into the soil. To directly assess rice N uptake from stylo mulch, ^15^N-enriched stylo mulch was produced during the 2011–2012 fallow period in designated ILT micro plots. This labelled mulch replaced non-labelled stylo mulch in micro plots.

In total, 12 DLT micro plots were established (3 treatments × 4 replicates).

*^15^N soil labelling – ILT*

Soil labelling was achieved by applying 60 atom% ^15^N-enriched (NH_4_)_2_SO_4_ at 9 kg N ha^-^¹. Simultaneously, sucrose was applied at 90 kg C ha^-^¹ to promote uniform soil N labelling through microbial turnover (Hood et al. 2000). The ^15^N solution was applied to the soil surface between stylo plants, following the same method as in the DLT. To minimize leaching, the application was split between December 2011 and February 2012 (Douxchamps et al. 2011). Pre-labelling the soil allowed equilibrium within labile N pools and minimized substitution effects during subsequent fertilizer N uptake measurements (Hood 2001). This also enabled the production of ^15^N-labeled stylo mulch for DLT (Bosshard et al. 2009).

To verify ILT results for FYM, the ILT approach was also applied to mineral fertilizer and stylo mulch, totalling 56 ILT micro plots (2 × 7 treatments × 4 replicates).

At rice harvest (April 2013), soil ^15^N enrichment in control micro plots was highest in the 0–0.1 m layer (0.200–0.350 atom% ¹^5^N excess) and decreased with depth (Fig. C, supplementary material).

| 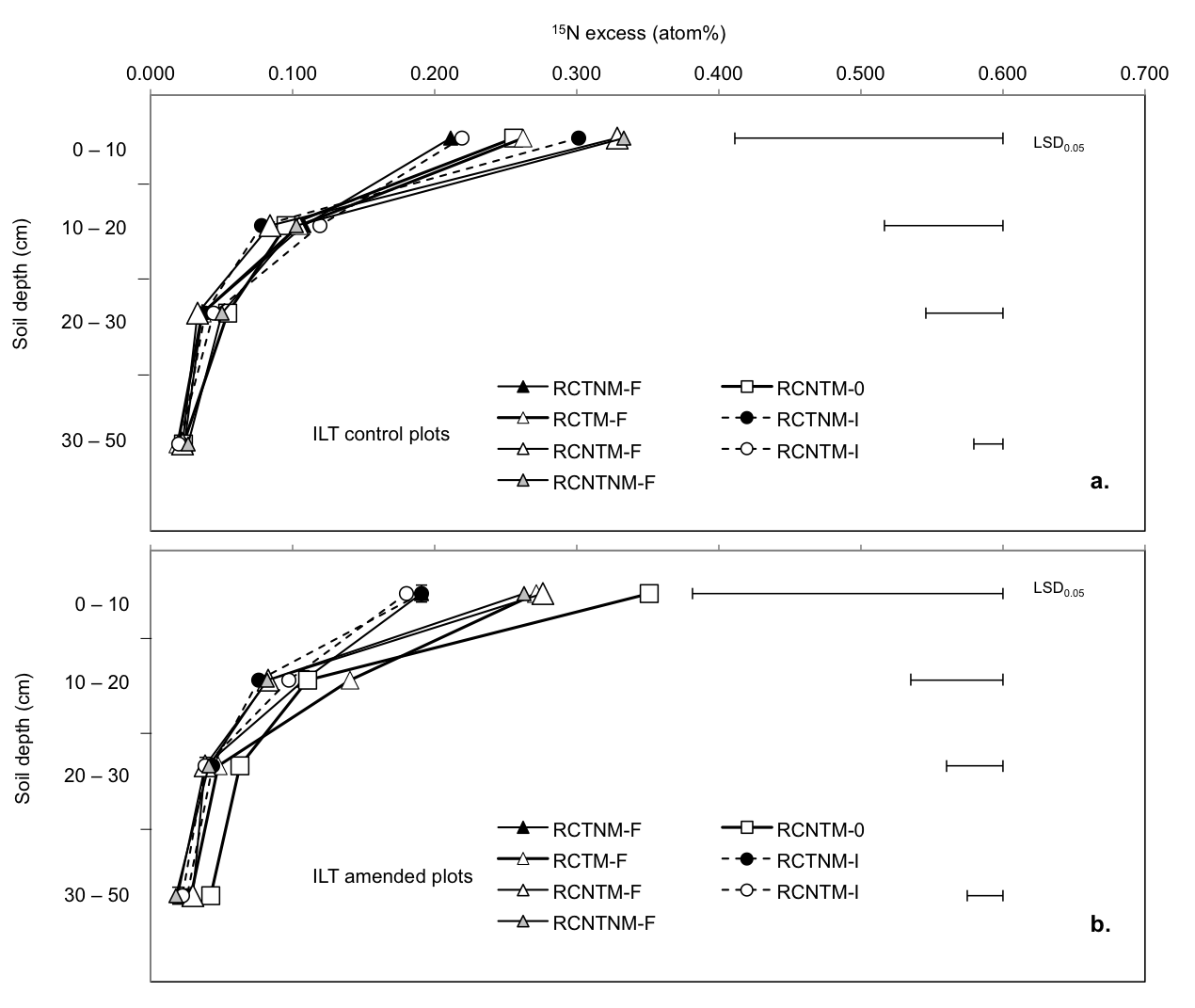 |
| --- |
| **Fig. C** Vertical soil ^15^N enrichment in control and respective amended micro plots for the indirect labelling approach (ILT) measured at rice harvest, at the end of season three (April 2013) in the field trial installed at the study site (Ivory, Madagascar). Error bars show LSD (*p* < 0.05) per sampling depth (*n*=4) |

| 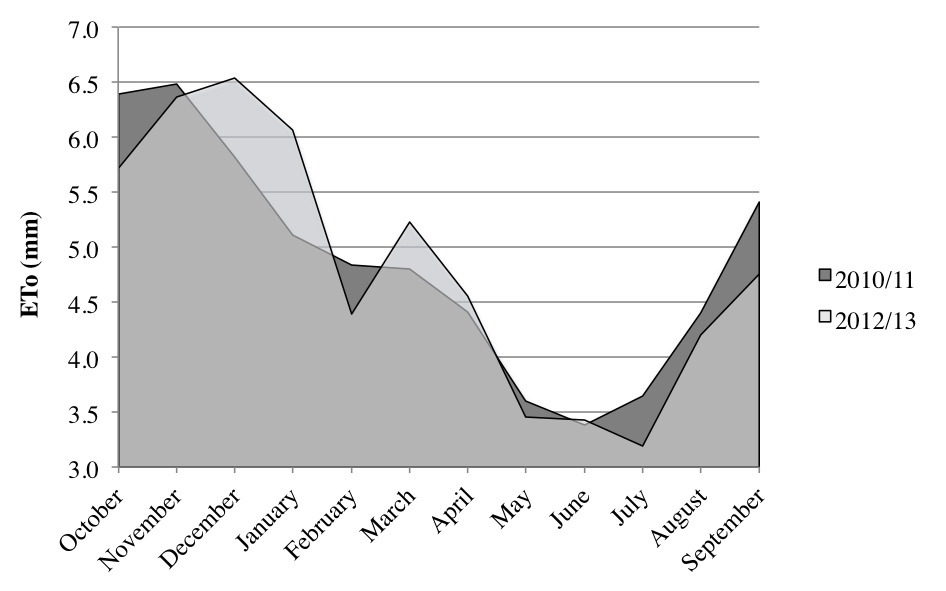 |
| --- |
| **Fig. D** The potential evapotranspiration (ETo) in the initial (2010/11) and the second (2012/13) cropping season in the field trial installed at the study site (Ivory, Madagascar) |

| **Table A** Rice dry matter yield and N concentration in core treatments (RCTNM-0, RCNTM-0, RCTNM-F, RCNTM-F, RCNTM-I and RCTNM-I) and the mono-crop treatment (MCTNM-0) prior and after the fallow season | | | | | | | | | | | | | | | | | | |
| --- | --- | --- | --- | --- | --- | --- | --- | --- | --- | --- | --- | --- | --- | --- | --- | --- | --- | --- |
| Cropping season |  |  | 1 (2010-11) | | | | | | |  | 2 (2012-13) | | | | | | |  |
| Plot |  |  | Main | | | | | | |  | Main | | | | | | |  |
|  |  |  | ^b^GY | SY | ^c^RY |  | GNC | SNC | RNC |  | ^b^GY | SY | ^c^RY |  | GNC | SNC | RNC |  |
| ^a^Management |  |  | g DM m^-2^ | | |  | g N kg^-1^ DM | | |  | g DM m^-2^ | | |  | g N kg^-1^ DM | | |  |
| Fertilizer (FM) |  |  |  |  |  |  |  |  |  |  |  |  |  |  |  |  |  |  |
| 0 |  |  | 258 | 179 | 128 |  | 13.9 | 5.5 | 8.3 |  | 290 | 303* | 84* |  | 11.4* | 5.0 | 13.3* |  |
| F |  |  | 313 | 202 | 137 |  | 14.7 | 4.8 | 8.4 |  | 377 | 318* | 91* |  | 10.6* | 4.1 | 12.2* |  |
| I |  |  | 441 | 329 | 170 |  | 14.6 | 6.7 | 8.9 |  | 429 | 404 | 103* |  | 14.2 | 5.3* | 13.5* |  |
| *SED* |  |  | *22.3* | *26.4* | *12.8* |  | *0.54* | *0.25* | *0.45* |  | *34.2* | *46.5* | *6.2* |  | *0.42* | *0.40* | *0.56* |  |
| *LSD_0.05_* |  |  | *60.1* | *71.1* | *34.5* |  | *1.45* | *0.66* | *1.21* |  | *92.2* | *125.2* | *16.6* |  | *1.14* | *1.08* | *1.51* |  |
| ^d^Soil + stylo mulch (SRM) | | |  |  |  |  |  |  |  |  |  |  |  |  |  |  |  |  |
| TNM |  |  | 343 | 243 | 149 |  | 14.5 | 5.7 | 8.7 |  | 324 | 296 | 114* |  | 12.0* | 4.8 | 13.9* |  |
| NTM |  |  | 331 | 231 | 142 |  | 14.3 | 5.6 | 8.5 |  | 408 | 388* | 71* |  | 12.2* | 4.8 | 12.1* |  |
| *SED* |  |  | *18.2* | *21.6* | *10.5* |  | *0.44* | *0.20* | *0.37* |  | *27.9* | *38.0* | *5.0* |  | *0.34* | *0.33* | *0.46* |  |
| *LSD_0.05_* |  |  | *38.9* | *46.0* | *22.3* |  | *0.94* | *0.43* | *0.78* |  | *59.6* | *80.9* | *10.7* |  | *0.73* | *0.70* | *0.97* |  |
| *Overall mean* |  |  | *337* | *237* | *145* |  | *14.4* | *5.7* | *8.6* |  | *366* | *342* | *92* |  | *12.1* | *4.8* | *13.0* |  |
| ^e^MCTNM-0 |  |  | 256 (78.2) | 151 (39.5) | *nd* |  | 14.2 (0.45) | 5.2 (0.13) | *nd* |  | 189 (61.5) | 153 (58.1) | *nd* |  | 12.2* (0.35) | 6.4 (0.13) | *nd* |  |
| *t – test (p)* |  |  | *ns* | *ns* | *na* |  | *ns* | *ns* | *na* |  | *** | *** | *na* |  | *ns* | *ns* | *na* |  |
| *Source of variation* |  | *df* |  |  |  |  |  |  |  |  |  |  |  |  |  |  |  |  |
| Block |  | 3 | ** | ** | * |  | *ns* | *ns* | ** |  | *ns* | *ns* | ** |  | *ns* | *ns* | *ns* |  |
| FM |  | 2 | *** | ** | * |  | *ns* | *** | *ns* |  | ** | *ns* | * |  | *** | *** | *ns* |  |
| SRM |  | 1 | *ns* | *ns* | *ns* |  | *ns* | *ns* | *ns* |  | ** | *** | *** |  | *ns* | *ns* | * |  |
| FM x SRM |  | 2 | *ns* | *ns* | *ns* |  | *ns* | *ns* | *ns* |  | *ns* | *ns* | *ns* |  | *ns* | ns | ns |  |
| Means, *n* = 8 (FM), *n* = 12 (SRM); Not significant (*ns*); * significant at the 0.05 level, ** significant at the 0.01 level, *** significant at the 0.001 level*;* standard error of difference (SED); Not applicable (*na*)*,* Not determined (*nd*) | | | | | | | | | | | | | | | | | | |
| Asterix* in rows indicate significant (t-test, *p* < 0.05) differences for the respective characteristics between cropping seasons | | | | | | | | | | | | | | | | | | |
| ^a^No fertilizer = 0 and FYM = F and NPK + urea = I; Tillage + No-Mulch = TNM, No-Tillage + Mulch = NTM | | | | | | | | | | | | | | | | | | |
| ^b^Paddy rice yield at 14% moisture content | | | | | | | | | | | | | | | | | | |
| ^c^Root dry matter (0-0.3 m soil depth) | | | | | | | | | | | | | | | | | | |
| ^d^Soils in all plots were tilled in cropping season 1. Statistical analysis was conducted to test for initial treatment differences, irrespective of SRM. | | | | | | | | | | | | | | | | | | |
| ^e^Results for the MCTNM-0 treatment were not included in the ANOVA. Shown are the mean (± standard deviation) and the t-test probabilities of significant difference to the mean of the RC-0 treatments (RCTNM-0, RCNTM-0) | | | | | | | | | | | | | | | | | | |
| Rice grain (GY), straw (SY) and root (RY) dry matter (DM) yield and grain (GNC), straw (SNC) and root (RNC) N concentration | | | | | | | | | | | | | | | | | | |

| **Table B** Rice dry matter yield, N concentration, N uptake and utilization efficiency in FYM-amended treatments (RCTNM-F, RCNTM-F, RCTM-F, RCNTNM-F) after the fallow season | | | | | | | | | | | | |
| --- | --- | --- | --- | --- | --- | --- | --- | --- | --- | --- | --- | --- |
| Cropping season |  | 2 (2012-13) | | | | | | | | | | |
| Plot |  | Main | | | | | | | | | | |
|  |  | ^b^GY | SY |  | GNC | SNC |  | GNU | SNU |  | ^c^pNUE |  |
| ^a^Management |  | g DM m^-2^ | |  | g N kg^-1^ DM | |  | g N m^-2^ | |  | g DM  g^-1^ N |  |
| Soil (SM) |  |  |  |  |  |  |  |  |  |  |  |  |
| T |  | 353 | 314 |  | 11.3 | 3.8 |  | 4.0 | 1.2 |  | 69 |  |
| NT |  | 384 | 337 |  | 10.9 | 4.2 |  | 4.2 | 1.4 |  | 69 |  |
| Stylo mulch (RM) |  |  |  |  |  |  |  |  |  |  |  |  |
| NM |  | 325 | 306 |  | 10.2 | 3.8 |  | 3.3 | 1.2 |  | 73 |  |
| M |  | 412 | 345 |  | 11.9 | 4.2 |  | 4.9 | 1.4 |  | 65 |  |
| *SED* |  | *23.6* | *33.7* |  | *0.28* | *0.38* |  | *0.36* | *0.30* |  | *3.1* |  |
| *LSD_0.05_* |  | *53.5* | *76.2* |  | *0.64* | *0.86* |  | *0.81* | *0.67* |  | *7.0* |  |
| *Overall mean* |  | *369* | *325* |  | *11.1* | *4.0* |  | *4.1* | *1.3* |  | *69* |  |
| *Source of variation* | *df* |  |  |  |  |  |  |  |  |  |  |  |
| Block | 3 | *ns* | *** |  | **** | *ns* |  | *ns* | *ns* |  | ** |  |
| SM | 1 | *ns* | *ns* |  | *ns* | *ns* |  | *ns* | *ns* |  | *ns* |  |
| RM | 1 | * | *ns* |  | **** | *ns* |  | * | *ns* |  | *ns* |  |
| SM x RM | 1 | *ns* | *ns* |  | ** | *ns* |  | *ns* | *ns* |  | *ns* |  |
| Means, *n* = 8 (SM), *n* = 8 (RM); Not significant (*ns*); * significant at the 0.05 level, ** significant at the 0.01 level, *** significant at the 0.001 level*;* standard error of difference (SED) | | | | | | | | | | | | |
| ^a^Tillage = T, No tillage = NT; No stylo mulch = NM, Stylo mulch = M | | | | | | | | | | | | |
| ^b^Paddy rice yield at 14% moisture content | | | | | | | | | | | | |
| ^c^GY / TNU | | | | | | | | | | | | |
| Rice grain (GY) and straw (SY) dry matter (DM) yield, grain (GNC) and straw (SNC) N concentration, grain (GNU) and straw (SNU) N uptake, and physiological N utilization efficiency (pNUE) | | | | | | | | | | | | |

| **Table C** Rice N uptake and utilization efficiency in core treatments (RCTNM-0, RCNTM-0, RCTNM-F, RCNTM-F, RCNTM-I and RCTNM-I) and the mono-crop treatment (MCTNM-0) prior and after the fallow season | | | | | | | | | | | | | |
| --- | --- | --- | --- | --- | --- | --- | --- | --- | --- | --- | --- | --- | --- |
| Cropping season |  |  |  | 1 (2010-11) | |  | | 2 (2012-13) | | |  | |  |
| Plot |  |  |  | Main | |  | | Main | | |  | |  |
|  |  |  | GNU | SNU | ^b^RNU | |  | ^e^pNUE | GNU | SNU | ^b^RNU | ^c^pNUE |  |
| ^a^Management |  |  |  | g N m^-2^ |  | | g DM g^-1^ N | | g N m^-2^ |  | g DM g^-1^ N | | |
| Fertilizer (FM) |  |  |  |  |  | |  |  |  |  |  |  |  |
| 0 |  |  | 3.6 | 1.0 | 1.1 | |  | 57 | 3.3 | 1.5* | 1.1 | 61 |  |
| F |  |  | 4.6 | 1.0 | 1.1 | |  | 56 | 4.1 | 1.3 | 1.1 | 72* |  |
| I |  |  | 6.4 | 2.2 | 1.5 | |  | 51 | 6.1 | 2.2 | 1.4 | 52 |  |
| *SED* |  |  | *0.37* | *0.20* | *0.12* | |  | *1.7* | *0.46* | *0.22* | *0.11* | *3.8* |  |
| *LSD_0.05_* |  |  | *0.99* | *0.55* | *0.33* | |  | *4.7* | *1.23* | *0.60* | *0.28* | *10.2* |  |
| ^d^Soil + stylo mulch (SRM) | | |  |  |  | |  |  |  |  |  |  |  |
| TNM |  |  | 5.0 | 1.5 | 1.3 | |  | 54 | 3.9 | 1.4 | 1.6* | 63 |  |
| NTM |  |  | 4.8 | 1.3 | 1.2 | |  | 55 | 5.0 | 1.9 | 0.9* | 60 |  |
| *SED* |  |  | *0.30* | *0.17* | *0.10* | |  | *1.4* | *0.37* | *0.18* | *0.09* | *3.1* |  |
| *LSD_0.05_* |  |  | *0.64* | *0.35* | *0.22* | |  | *3.0* | *0.80* | *0.39* | *0.18* | *6.6* |  |
| *Overall mean* |  |  | *4.9* | *1.4* | *1.2* | |  | *55* | *4.5* | *1.6* | *1.2* | *62* |  |
| ^e^MCTNM-0 |  |  | 3.6 (1.08) | 0.8 (0.19) | *nd* | |  | 58 (1.6) | 2.3 (0.78) | 1.0 (0.40) | *nd* | 58 (3.7) |  |
| *t – test (p)* |  |  | *ns* | *ns* | *na* | |  | *ns* | *ns* | *ns* | *na* | *ns* |  |
| *Source of variation* |  | df |  |  |  | |  |  |  |  |  |  |  |
| Block |  | 3 | **** | ** | ** | |  | *ns* | *ns* | *ns* | ** | *ns* |  |
| FM |  | 2 | *** | ** | * | |  | * | *** | *** | *ns* | *** |  |
| SRM |  | 1 | *ns* | *ns* | *ns* | |  | *ns* | *** | * | *** | *ns* |  |
| FM x SRM |  | 2 | *ns* | *ns* | *ns* | |  | *ns* | *ns* | *ns* | *ns* | *ns* |  |
| Means, *n* = 8 (FM), *n* = 12 (SRM); Not significant (*ns*); * significant at the 0.05 level, ** significant at the 0.01 level, *** significant at the 0.001 level*;* standard error of difference (SED); Not applicable (*na*)*,* Not determined (*nd*) | | | | | | | | | | | | | |
| Asterix* in rows indicate significant (t-test, *p* < 0.05) differences for the respective characteristics between cropping seasons | | | | | | | | | | | | | |
| ^a^Tillage + No-Mulch = TNM, NTM = No-Tillage + Mulch; no fertilizer = 0 and FYM = F and NPK + urea = I | | | | | | | | | | | | | |
| ^b^Root dry matter (0-0.3 m soil depth) | | | | | | | | | | | | | |
| ^c^GY / TNU | | | | | | | | | | | | | |
| ^d^Soils in all plots were tilled in cropping season 1. Statistical analysis was conducted to test for initial treatment differences, irrespective of SRM. | | | | | | | | | | | | | |
| ^e^Results for the MCTNM-0 treatment were not included in the ANOVA. Shown are the mean (± standard deviation) and the t-test probabilities of significant difference to the mean of the RC-0 treatments (RCTNM-0, RCNTM-0) | | | | | | | | | | | | | |
| Rice grain (GNU), straw (SNU), root (RNU) N uptake and physiological utilization efficiency (pNUE) | | | | | | | | | | | | | |

| **Table D** Recovery of stylo mulch, FYM, and mineral fertilizer N assessed with ILT and DLT techniques (FNRec) and assessed by the apparent recovery method (ANREc) in rice plant parts (straw, grain) at harvest | | | | | | | | | | | | | |
| --- | --- | --- | --- | --- | --- | --- | --- | --- | --- | --- | --- | --- | --- |
| Cropping season | | | 3 (2012-13) | | | | | | | | | | |
| Plot | | | Micro | | | | | | | | | | |
|  | | | ANREc or FNRec (%) | | | | | | | | | | |
|  |  |  | Straw | | |  | Grain | | |  | ^a^Total | | |
| ^b^Treatment | Fertilizer |  | ANRec | DLT | ILT |  | ANREc | DLT | ILT |  | ANREc | DLT | ILT |
| RCNTM-0 | Stylo mulch |  | *^c^(-2)* | 1 | *^c^(-1)* |  | 0 | 4 | 0 |  | *^c^(-2)* | 5 | *^c^(0)* |
| RCTNM-F | FYM |  | 2 | - | 4 |  | 11 | - | 14 |  | 14 | - | 18 |
| RCNTM-F | FYM |  | 0 | - | *^c^(-1)* |  | 1 | - | *^c^(-2)* |  | 2 | - | *^c^(-2)* |
| RCTM-F | FYM |  | 2 | - | *^c^(0)* |  | 9 | - | 3 |  | 11 | - | *^c^(-2)* |
| RCNTNM-F | FYM |  | 2 | - | 4 |  | 10 | - | 13 |  | 12 | - | 18 |
| RCTNM-I | NPK + urea | | 11 | 8 | 12 |  | 49 | 40 | 44 |  | 60 | 48 | 56 |
| RCNTM-I | NPK + urea | | 14 | 9 | 6 |  | 49 | 50 | 36 |  | 63 | 59 | 42 |
| *SED* |  |  | *2.8* | *1.3* | *2.7* |  | *10.9* | *4.0* | *8.2* |  | *13.3* | *5.0* | *9.9* |
| *LSD_0.05_* | *df* |  | *9.8* | *4.1* | *9.5* |  | *38.4* | *13.2* | *28.9* |  | *47.0* | *16.6* | *34.9* |
| *Source of variation* |  |  |  |  |  |  |  |  |  |  |  |  |  |
| Block | *3* |  | *ns* | *ns* | *ns* |  | *** | *ns* | *ns* |  | *** | *ns* | *ns* |
| Treatment | 6 |  | *** | ** | ** |  | *** | *** | *** |  | *** | *** | *** |
| Means, *n* = 4; Not significant (*ns*); * significant at the 0.05 level, ** significant at the .01 level, *** significant at the .001 level*;* standard error of difference (SED) | | | | | | | | | | | | | |
| ^a^Shoot = grain + straw | | | | | | | | | | | | | |
| ^b^Relay-cropping = RC; Tillage = T, No-tillage = NT, Stylo mulch = M, No stylo mulch = NM; no fertilizer = 0, FYM = F and NPK + urea = I | | | | | | | | | | | | | |
| ^c^method failed (recovery estimates obtained using the ILT or ANRec due to negative Ndff values) | | | | | | | | | | | | | |

**References**

Bosshard, C, Sorensen, P, Frossard, E, Dubois, D, Mader, P, Nanzer, S, Oberson, A (2009) Nitrogen use efficiency of N-15-labelled sheep manure and mineral fertiliser applied to microplots in long-term organic and conventional cropping systems. Nutr Cycl Agroecosys 83, 271-287. <https://doi.org/10.1007/S10705-008-9218-7>

Douxchamps, S, Frossard, E, Bernasconi, SM, van der Hoek, R, Schmidt, A, Rao, IM, Oberson, A (2011) Nitrogen recoveries from organic amendments in crop and soil assessed by isotope techniques under tropical field conditions. Plant Soil 341, 179-192. <https://doi.org/10.1007/S11104-010-0633-6>

Hood, R (2001) Evaluation of a new approach to the nitrogen-15 isotope dilution technique, to estimate crop N uptake from organic residues in the field. Biol Fert Soils 34, 156-161. <https://doi.org/10.1007/s003740100388>

Hood, R, Merckx, R, Jensen, ES, Powlson, D, Matijevic, M, Hardarson, G (2000) Estimating crop N uptake from organic residues using a new approach to the N-15 isotope dilution technique. Plant Soil 223, 33-44. <https://doi.org/10.1023/A:1004789103949>
